# Intrapatient Intermetastatic Heterogeneity Determined by Triple-Tracer PET Imaging in mCRPC Patients and Correlation to Survival: The 3TMPO Cohort Study

**DOI:** 10.2967/jnumed.124.268020

**Published:** 2024-11

**Authors:** Frédéric Pouliot, Fred Saad, Etienne Rousseau, Patrick O. Richard, Atefeh Zamanian, Stephan Probst, Éric Lévesque, Vincent Castonguay, Nicolas Marcoux, Michele Lodde, Daniel Juneau, Zineb Hamilou, Jean-Baptiste Lattouf, François-Alexandre Buteau, Michel Pavic, Jean-François Castilloux, Bertrand Neveu, Guillaume F. Bouvet, Catherine Allard, Amélie Tétu, Brigitte Guérin, Jean-Mathieu Beauregard, for the 3TMPO Investigators

**Affiliations:** 1 Oncology Axis, CHU de Québec–Université Laval Research Center, Quebec City, Quebec, Canada;; 2 Division of Urology, Department of Surgery, Université Laval, Quebec City, Quebec, Canada;; 3 Division of Urology, Department of Surgery, Centre Hospitalier de l’Université de Montréal, Université de Montréal, Montréal, Quebec, Canada;; 4 Department of Nuclear Medicine and Radiobiology, Université de Sherbrooke, Sherbrooke, Quebec, Canada;; 5 Sherbrooke Molecular Imaging Centre, Centre de Recherche du Centre Hospitalier Universitaire de Sherbrooke, Sherbrooke, Quebec, Canada;; 6 Division of Urology, Department of Surgery, Centre Hospitalier Universitaire de Sherbrooke and Centre de Recherche du Centre Hospitalier Universitaire de Sherbrooke, Sherbrooke, Quebec, Canada;; 7 Department of Radiology and Nuclear Medicine, and Cancer Research Centre, Université Laval, Quebec City, Quebec, Canada;; 8 Division of Nuclear Medicine, Faculty of Medicine, Sir Mortimer B. Davis–Jewish General Hospital, McGill University, Montréal, Quebec, Canada;; 9 Division of Hemato-Oncology, Department of Medicine, CHU de Québec–Université Laval, Quebec City, Quebec, Canada;; 10 Surgery Urology Department, CHU de Québec–Université Laval, Quebec City, Quebec, Canada;; 11 Nuclear Medicine, Centre Hospitalier de l’Université de Montréal, Montreal, Quebec, Canada;; 12 Hemato-Oncology, Centre Hospitalier de l’Université de Montréal, Montreal, Quebec, Canada;; 13 Division of Nuclear Medicine, Department of Medical Imaging, CHU de Québec–Université Laval, Quebec City, Quebec, Canada;; 14 Medical Oncology, Centre Intégré Universitaire de Santé et Services Sociaux de l’Estrie–Centre Hospitalier Universitaire de Sherbrooke, Sherbrooke, Quebec, Canada; and; 15 Unité de Recherche Clinique et Épidémiologique, Centre de Recherche du Centre Hospitalier Universitaire de Sherbrooke, Sherbrooke, Quebec, Canada

**Keywords:** metastatic castration-resistant prostate cancer, molecular imaging, PET, intrapatient intermetastatic heterogeneity, IIH

## Abstract

Intrapatient intermetastatic heterogeneity (IIH) has been demonstrated in metastatic castration-resistant prostate cancer (mCRPC) patients and is of the utmost importance for radiopharmaceutical therapy (RPT) eligibility. This study was designed to determine the prevalence of IIH and RPT eligibility in mCRPC patients through a triple-tracer PET imaging strategy. **Methods:** This was a multisite prospective observational study in which mCRPC patients underwent both ^18^F-FDG and ^68^Ga-prostate-specific membrane antigen (PSMA)–617 PET/CT scans. A third scan with ^68^Ga-DOTATATE, a potential biomarker of neuroendocrine differentiation, was performed if an ^18^F-FDG–positive/^68^Ga-PSMA–negative lesion was found. Per-tracer lesion positivity was defined as having an uptake at least 50% above that of the liver. IIH prevalence was defined as the percentage of participants having at least 2 lesions with discordant features on multitracer PET. **Results:** IIH was observed in 81 patients (82.7%), and at least 1 ^18^F-FDG–positive/^68^Ga-PSMA–negative lesion was found in 45 patients (45.9%). Of the 37 participants who also underwent ^68^Ga-DOTATATE PET/CT, 6 (16.2%) had at least 1 ^68^Ga-DOTATATE–positive lesion. In total, 12 different combinations of lesion imaging phenotypes were observed. On the basis of our prespecified criteria, 52 (53.1%) participants were determined to be eligible for PSMA RPT, but none for DOTATATE RPT. Patients with IIH had a significantly shorter median overall survival than patients without IIH (9.5 mo vs. not reached; log-rank *P* = 0.03; hazard ratio, 2.7; 95% CI, 1.1–6.8). **Conclusion:** Most mCRPC patients showed IIH, which was associated with shorter overall survival. On the basis of a triple-tracer PET approach, multiple phenotypic combinations were found. Correlation of these imaging phenotypes with genomics and treatment response will be relevant for precision medicine.

## Visual Abstract

**Figure:**
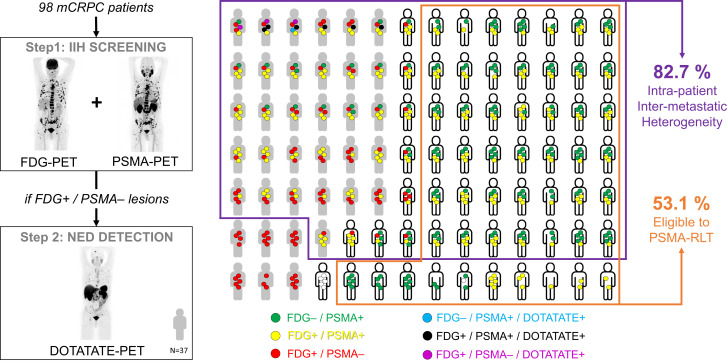

Patients now have the opportunity to undergo several lines of systemic therapies targeting nonredundant cancer pathways. Although these therapies have translated into better survival, cancer phenotypic changes secondary to successive drug exposure (i.e., phenotypic plasticity) have led to challenges in monitoring cancer treatment resistance, defining progression and therapeutic decision-making (1,2). Among these challenges, the intrapatient intermetastatic heterogeneous cancer evolution is an example of phenotypic plasticity. Intrapatient intermetastatic heterogeneity (IIH) implies that, in a single patient, various clones of cancer may be present in different metastases, each potentially harboring differential drug sensitivity (3). Most studies have been performed through rapid autopsy or have been limited to the analysis of only a few metastases.

Castration-resistant prostate cancer (CRPC) can evolve either as classic adenocarcinoma, androgen receptor–indifferent carcinoma, or neuroendocrine CRPC (4,5). Neuroendocrine CRPC is an aggressive subset of castration-resistant tumors that exhibit neuroendocrine differentiation (NED) pathologic features on biopsy, including somatostatin receptor, chromogranin, and synaptophysin expression (6,7). Metastatic CRPC (mCRPC) may evolve as an intrapatient heterogeneous disease based on genomic analyses of intrapatient metastatic tissues or imaging. Using functional PET imaging, our group and others have shown that as many as 40% of metastatic prostate cancer patients exhibit evidence of IIH (8–12). This can manifest, in a given patient, as discordant tracer uptake or histopathology among different metastases as well as a heterogeneous response to systemic therapies. However, the true prevalence of IIH in mCRPC is unknown.

To answer this question, multitracer molecular imaging offers a unique way to determine the prevalence of IIH or CRPC NED in prostate cancer. Indeed, specific PET tracers can noninvasively characterize adenocarcinoma by targeting the prostate-specific membrane antigen (PSMA; e.g.,^68^Ga-PSMA-617) or neuroendocrine CRPC via the somatostatin receptor (^68^Ga-DOTATATE), offering new tools for in vivo visualization of IIH (13). Although ^68^Ga-PSMA PET/CT is highly sensitive for detecting metastases in mCRPC patients, this tracer may miss CRPC NED without PSMA expression. ^18^F-FDG uptake tends to be low in low-grade or indolent prostate cancer but progressively increases as these cells become more rapidly proliferative, such as in mCRPC or CRPC NED (14). Therefore, ^18^F-FDG PET in combination with ^68^Ga-PSMA PET could be helpful to detect IIH, as well as to screen for potential CRPC NED. In this prospective study, we used a stepwise triple-tracer molecular imaging approach to determine the prevalence of IIH and its associated impact on overall survival (OS) in progressive mCRPC.

## MATERIALS AND METHODS

### Study Design and Participants

This was a multicenter prospective observational cohort study conducted at 5 Canadian academic hospital centers (Supplemental Table 1; supplemental materials are available at http://jnm.snmjournals.org). The study was approved by a central research ethics board, and all subjects signed an informed consent form. The protocol was also registered on Clinicaltrials.gov (NCT04000776) and published (15). The use of 2 investigational radiopharmaceuticals, ^68^Ga-PSMA-617 and ^68^Ga-DOTATATE (with cyclotron-produced ^68^Ga), was approved by Health Canada for research purposes (16,17).

Eligible participants were male adults (≥18 y old) with a history of pathologically proven adenocarcinoma of the prostate and with at least 3 active metastases on conventional imaging and evidence of biochemical or radiographic progression under continuous androgen deprivation (Supplemental Table 2). Baseline data included medical and prostate cancer history, blood test results, and survival at the last visit (12 ± 1 mo after the first PET scan).

### PET/CT Imaging

All participants underwent ^18^F-FDG and ^68^Ga-PSMA-617 PET/CT within 10 d. A third PET/CT scan with ^68^Ga-DOTATATE was performed within 10 d from the second PET scan for patients who were found to have at least 1 ^18^F-FDG–positive (+)/^68^Ga-PSMA–negative (−) lesion. ^68^Ga-PSMA-617 (1.8–2.2 MBq/kg) and ^68^Ga-DOTATATE (2.2–4 MBq/kg) were used, with maximum doses of 300 MBq and 370 MBq, respectively. The ^18^F-FDG radiopharmaceutical is authorized for market and was used as per local protocols. PET acquisitions were performed from the vertex to the proximal thighs along with low-dose CT without contrast medium after an uptake period of 60 ± 5 min.

Patients were selected for ^68^Ga-DOTATATE PET/CT on the basis of live ^18^F-FDG and ^68^Ga-PSMA-617 PET/CT review by a central reviewer, who searched for the presence of at least 1 ^18^F-FDG+/^68^Ga-PSMA− lesion using GOLD software (Hermes Medical Solutions) and the SUV tool, but without full tumor segmentation. A lesion was positive for a given tracer when its SUV_peak_ ratio (i.e., the lesion SUV_peak_ divided by the liver SUV_mean_) was at least 1.5.

For the final analysis, using MIM Encore software (MIM Software Inc.), all lesions were individually segmented as follows: first, a threshold of 1.5 times the liver SUV_mean_ (measured with a 3-cm spheric volume of interest [VOI]) was applied to automatically delineate all VOIs with significant activity. Second, VOIs smaller than 1 cm^3^ were discarded from the analysis. Third, VOIs representing physiologic or likely benign uptake were excluded (for ambiguous cases, a consensus was reached between the 2 central readers). Finally, each remaining VOI of contiguous uptake of any tracer was considered to represent 1 lesion. Using this approach, up to 10 lesions per compartment (i.e., nodes, bones, liver, or other; selecting the 10 hottest ones when >10) and ^18^F-FDG/^68^Ga-PSMA imaging phenotype (i.e., ^18^F-FDG+/^68^Ga-PSMA−, ^18^F-FDG+/^68^Ga-PSMA+, and ^18^F-FDG−/^68^Ga-PSMA+) were matched between the 2- or 3-tracer PET images, labeled, and recorded (i.e., up to 120 lesions per participant). SUV_peak_ ratio was computed for each lesion and each tracer.

### Endpoints

The prevalence of IIH was the primary endpoint and was defined as a participant’s having at least 2 lesions with different ^18^F-FDG/^68^Ga-PSMA/^68^Ga-DOTATATE imaging phenotypes. Secondary endpoints were, first, the proportion of participants with suspected CRPC NED on imaging, defined as having at least 1 ^68^Ga-DOTATATE+ lesion; second, the proportion of participants eligible for PSMA radiopharmaceutical therapy (RPT), defined as having at least 1 ^68^Ga-PSMA+ lesion and no ^18^F-FDG+/^68^Ga-PSMA− lesions; and third, the proportion of participants eligible for DOTATATE RPT, defined as having at least 1 ^68^Ga-DOTATATE+ lesion and no ^18^F-FDG+/^68^Ga-DOTATATE− lesions.

### Sample Size and Statistical Analysis

We calculated that a minimum of 81 men were needed to obtain ±10% precision with a 95% CI, considering a hypothesized prevalence of IIH of 30% or a minimum of 97 men for a prevalence of 50% for which the 95% CI would be the largest (9,18).

Continuous data were presented with mean and SD when the distribution was normal or with median and interquartile range when not normal. Frequencies and proportions were used to present categoric variables. Descriptive analyses were also used to present the prevalence of IIH, CRPC NED, and participants’ eligibility for PSMA or DOTATATE RPT (95% CI is based on the Clopper–Pearson exact binomial method). χ^2^ and Fisher exact tests were used to compare proportions between groups. The *t* test and Mann–Whitney test were used to compare continuous variables between groups. OS was calculated from the date of the first PET/CT scan to the date of death (of any cause). Patients who were still alive at the end of the follow-up were censored at the date of last follow-up to a maximum of 13 mo. Kaplan–Meier plots were used to graphically show the OS by group, and log-rank tests were performed to compare groups. In addition, a Cox proportional-hazards model was used to estimate the hazard ratio (HR) between groups. Median survivals and HRs were presented along with their 95% CI. A complete case analysis approach was used to handle missing values. All analyses were performed using R version 4.3.0. A result with a *P* value of less than 0.05 was considered statistically significant.

## RESULTS

### IIH Based on Dual ^18^F-FDG and ^68^Ga-PSMA PET/CT Imaging

**FIGURE 1.**
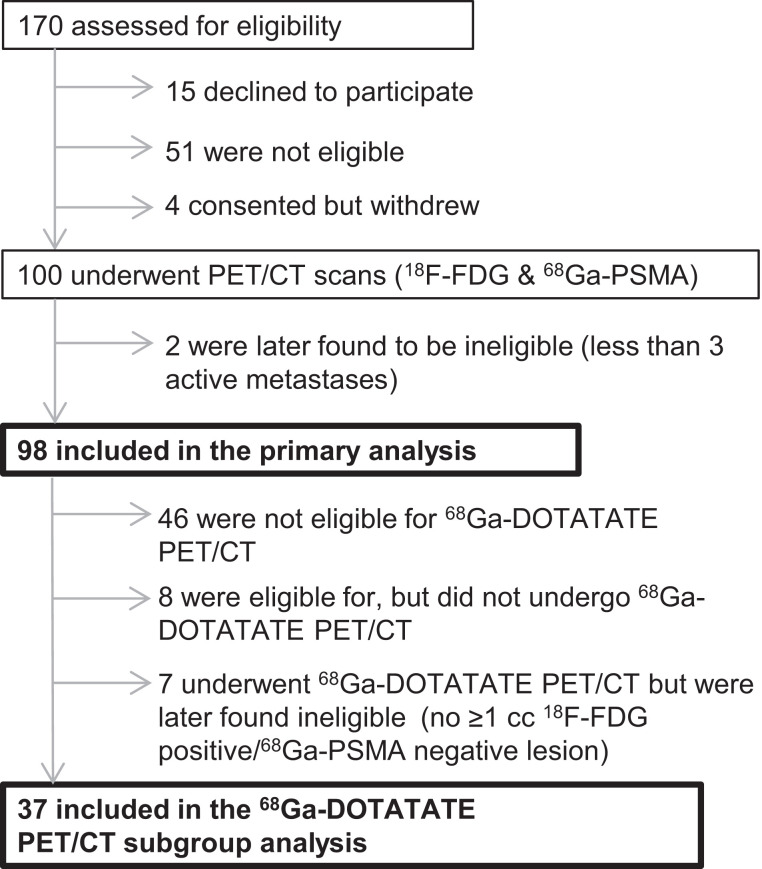
3TMPO flow diagram.

**FIGURE 2.**
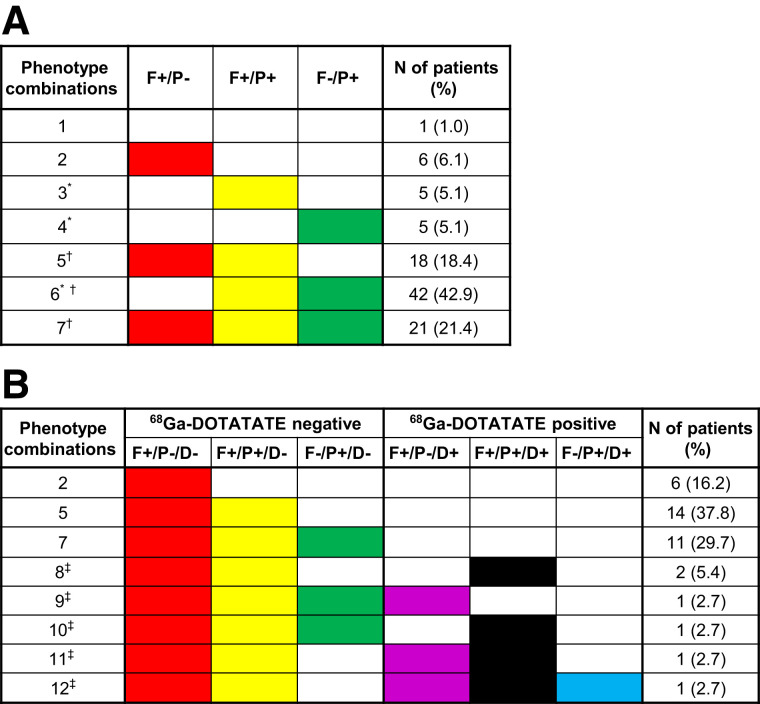
(A) Intrapatient intermetastatic phenotypes based on ^18^F-FDG (F) and ^68^Ga-PSMA (P) tracer PET/CT in 98 mCRPC patients. (B) Intrapatient intermetastatic imaging phenotypes based on ^18^F-FDG (F), ^68^Ga-PSMA (P), and ^68^Ga-DOTATATE (D) PET/CT in 37 mCRPC patients with ≥1 F+/P− lesion who underwent ^68^Ga-DOTATATE scanning. *Phenotypes eligible for PSMA RPT. ^†^Phenotypes with IIH. ^‡^Phenotypes with ≥1 D+ lesion.

In total, 98 participants were included in the final analyses (Fig. 1). Forty-four participants underwent ^68^Ga-DOTATATE PET/CT as an exploratory biomarker of CRPC NED, but only 37 participants were included in the subanalyses (Fig. 1). The use of the 3 radiopharmaceuticals was not related to any serious adverse events (Supplemental Table 3). Clinicopathologic characteristics of the ^18^F-FDG/^68^Ga-PSMA–imaged cohort (*n* = 98) are reported in Table 1. On the basis of prespecified ^18^F-FDG and ^68^Ga-PSMA PET/CT scan criteria for lesion positivity, the prevalence of IIH was 82.7% (81/98; Fig. 2A). Interestingly, no clinicopathologic factor besides prostate-specific antigen level was associated with the presence of IIH (Table 1). Forty-five (45.9%) had at least 1 ^18^F-FDG+/^68^Ga-PSMA− lesion (phenotype combinations 2, 5, and 7). Clinicopathologic factors that were significantly associated with the presence of at least 1 ^18^F-FDG+/^68^Ga-PSMA− lesion are presented in Table 2.

**TABLE 1.** Patients’ Characteristics at Baseline

Variable	All patients (*n* = 98)	With IIH (*n* = 81)*	Without IIH (*n* = 16)*	*P*
Age at consent (y)	69.2 ± 7.4	69.33 ± 7.39	68.81 ± 8.05	0.81^†^
Ethnicity (European descent)	95 (96.9)	78 (96.3)	16 (100.0)	1^‡^
ECOG^+^				
0	38 (39.2)	31 (79.5)	8 (20.5)	0.55^‡^
1	50 (51.5)	42 (87.5)	6 (12.5)	
2	9 (9.3)	7 (77.8)	2 (22.2)	
Baseline PSA (ng/mL)	51.1 (18.9–206.0)	62.27 (27.21–237.61)	15.76 (2.3–38.7)	0.00084^ǁ^
ISUP grade group at diagnosis^§^				
1	4 (4.3)	3 (75.0)	1 (25.0)	0.55^‡^
2	5 (5.4)	5 (100.0)	0 (0.0)	
3	12 (13.0)	8 (72.7)	3 (27.3)	
4	23 (25.0)	19 (82.6)	4 (17.4)	
5	48 (52.2)	42 (87.5)	6 (12.5)	
Number of metastases				
<5	17 (17.3)	33 (78.6)	9 (21.4)	0.19^‡^
5–9	27 (27.6)	22 (95.7)	1 (4.3)	
≥10	54 (55.1)	26 (81.3)	6 (18.7)	
Number of treatment lines^¶^				
0	8 (8.2)	5 (62.5)	3 (37.5)	0.42^‡^
1	20 (20.4)	17 (85.0)	3 (15.0)	
2	12 (12.2)	10 (83.3)	2 (16.7)	
>2	58 (59.2)	49 (86.0)	8 (14.0)	
Location of metastases				
Bone	93 (94.9)	78 (84.8)	14 (15.2)	0.19^‡^
Lymph nodes	61 (62.2)	54 (88.5)	7 (11.5)	0.15^‡^
Viscera	25 (25.5)	21 (84.0)	4 (16.0)	1^‡^

* Excludes patient who was ^18^F-FDG–negative and ^68^Ga-PSMA–negative.

† *t* test.

‡ Fisher test.

+ One missing datum.

§ Six missing data.

ǁ Wilcoxon signed-rank test.

¶ All lines including docetaxel during hormone-sensitive prostate cancer.

ECOG = Eastern Cooperative Oncology Group; PSA = prostate-specific antigen; ISUP = International Society of Urologic Pathologists.

Data are mean ± SD, median and interquartile range, or number and percentage.

**TABLE 2.** Comparison Between Patients with ≥1 ^18^F-FDG+/^68^Ga-PSMA− Lesion and Those Without (*n* = 97)*

Variable	≥1 ^18^F-FDG+/^68^Ga-PSMA− lesion (*n* = 45)	No ^18^F-FDG+/^68^Ga-PSMA− lesion (*n* = 52)	*P*
Age at consent (y)	68.44 ± 6.59	69.94 ± 8.14	0.32^†^
ECOG^‡^			1.00^§^
0	17 (45.9)	20 (54.1)	
1	24 (48.0)	26 (52.0)	
2	4 (44.4)	5 (55.6)	
Baseline PSA (μg/L)^‡^	42.7 (16.2–206.0)	58.2 (30.2–198.8)	0.27^ǁ^
ISUP grade group at diagnosis^¶^			0.28^§^
1	2 (50.0)	2 (50.0)	
2	3 (60.0)	2 (40.0)	
3	4 (36.4)	7 (63.6)	
4	7 (30.4)	16 (69.6)	
5	27 (56.3)	21 (43.8)	
Number of metastases			0.07^#^
<5	7 (41.2)	10 (58.8)	
5–9	8 (29.6)	19 (70.4)	
≥10	30 (56.6)	23 (43.4)	
Number of treatment lines**			0.03^§^
0	1 (12.5)	7 (87.5)	
1	7 (35.0)	13 (65.0)	
2	9 (75.0)	3 (25.0)	
>2	28 (49.1)	29 (50.9)	
Location of metastases			
Bone	42 (45.7)	50 (54.3)	0.66^§^
Lymph nodes	26 (42.6)	35 (57.4)	0.45^#^
Viscera	17 (68.0)	8 (32.0)	0.02^#^

* Excludes patient who was ^18^F-FDG−/^68^Ga-PSMA−.

† *t* test.

‡ One missing datum.

§ Fisher test.

ǁ Wilcoxon signed-rank test.

¶ Six missing data.

# χ^2^ test.

** All including docetaxel during hormone-sensitive prostate cancer.

ECOG = Eastern Cooperative Oncology Group; PSA = prostate-specific antigen; ISUP = International Society of Urologic Pathologists.

Data are mean ± SD, median and interquartile range, or number and percentage.

### IIH Based on Triple ^18^F-FDG, ^68^Ga-PSMA, and ^68^Ga-DOTATATE PET/CT Imaging

**FIGURE 3.**
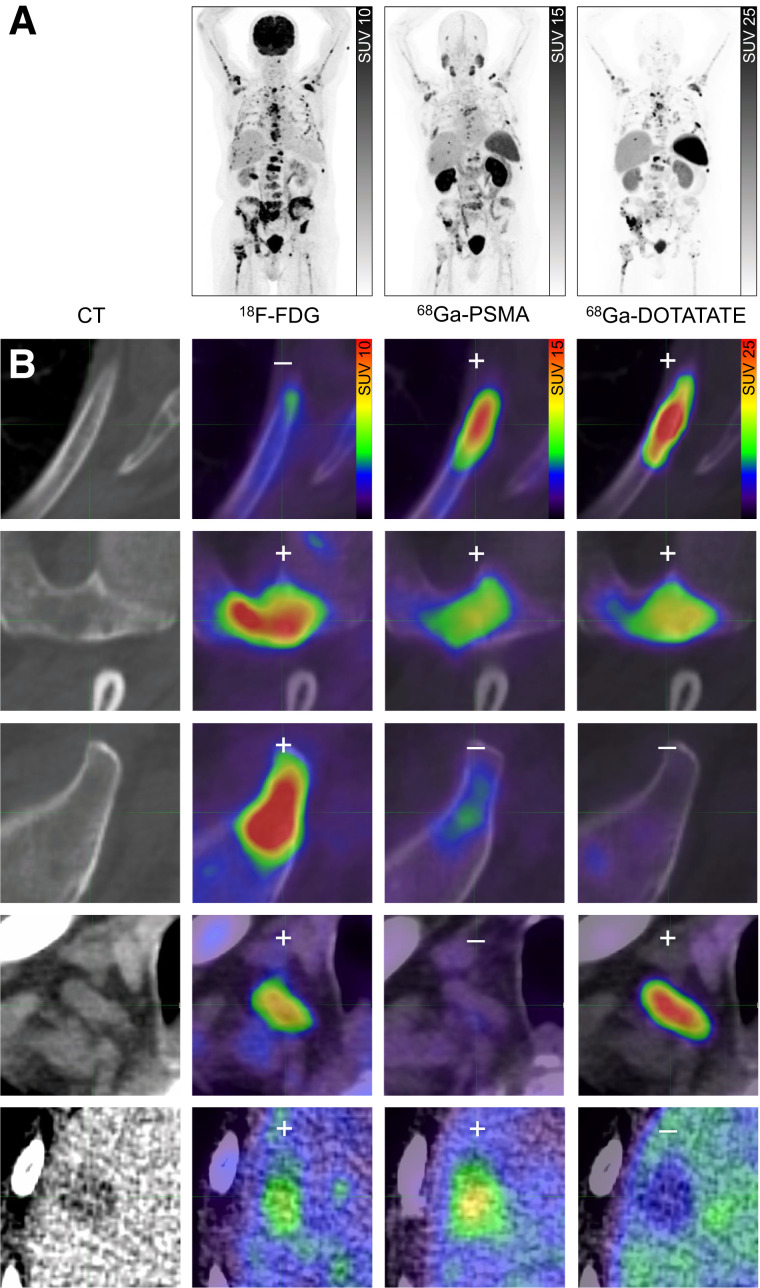
Patient with 5 different lesion phenotypes seen on triple-tracer PET/CT imaging. (A) Coregistered transaxial slices of whole-body maximum-intensity projection PET/CT showing multiple metastases with 3 different radiopharmaceuticals. (B) Discordant expression of 5 representative lesions: bone lesions of seventh left rib (first row) and left scapula (second row), lesions in left iliac (third row) and left supraclavicular (fourth row) lymph nodes, and lesion in right liver lobe (fifth row).

In participants who underwent ^68^Ga-DOTATATE PET/CT (*n* = 37), the prevalence of IIH was 83.8% (Fig. 2B). ^68^Ga-DOTATATE PET/CT was positive in 16.2% of this subgroup using the prespecified positivity criterion. The addition of ^68^Ga-DOTATATE PET/CT identified 5 additional phenotypic combinations. Among these combinations, 1 participant had 5 different phenotypes (phenotype combination 12; Figs. 2B and 3). Therefore, a total of 12 lesion phenotypic combinations was observed in the overall cohort when considering the ^68^Ga-DOTATATE PET/CT findings. Clinicopathologic factors associated with patients showing at least 1 ^68^Ga-DOTATATE+ lesion are presented in Table 3.

**TABLE 3.** Comparison Between Patients with ≥1 ^68^Ga-DOTATATE–Avid Lesion and Those Without (*n* = 37)*

Variable	≥1 ^68^Ga-DOTATATE–avid lesion (*n* = 6)	No ^68^Ga-DOTATATE–avid lesion (*n* = 31)	*P*
Age at consent (y)	71.67 ± 3.33	67.58 ± 6.62	0.04^†^
ECOG			
0	2 (14.3)	12 (85.7)	1^‡^
1	4 (18.2)	18 (81.8)	
2	0 (0.0)	1 (100.0)	
Baseline PSA (ng/mL)	122.8 (6.2–317.6)	51.1 (12.8–178.4)	0.92^§^
ISUP grade group at diagnostic^ǁ^			
1	0 (0.0)	2 (100.0)	0.65^‡^
2	1 (33.3)	2 (66.7)	
3	0 (0.0)	3 (100.0)	
4	0 (0.0)	6 (100.0)	
5	5 (23.8)	16 (76.2)	
Number of metastases			
<5	1 (16.7)	5 (83.3)	0.68^‡^
5–9	0 (0.0)	7 (100.0)	
≥10	5 (20.8)	19 (79.2)	
Number of treatment lines^¶^			
0	0 (0.0)	1 (100.0)	0.49^‡^
1	0 (0.0)	4 (100.0)	
2	3 (33.3)	6 (66.7)	
>2	3 (13.0)	20 (87.0)	
Location of metastases			
Bone	6 (17.6)	28 (82.4)	1^‡^
Lymph nodes	4 (20.0)	16 (80.0)	0.67^‡^
Viscera	5 (33.3)	10 (66.7)	0.03^‡^

* Includes 37 patients who underwent ^68^Ga-DOTATATE PET/CT.

† *t* test.

‡ Fisher test.

§ Wilcoxon signed-rank test.

ǁ Two missing data.

¶ All lines including docetaxel during hormone-sensitive prostate cancer.

ECOG = Eastern Cooperative Oncology Group; PSA = prostate-specific antigen; ISUP = International Society of Urologic Pathologists.

Data are mean ± SD, median and interquartile range, or number and percentage.

### Impact of IIH and Imaging Phenotypes on OS

**FIGURE 4.**
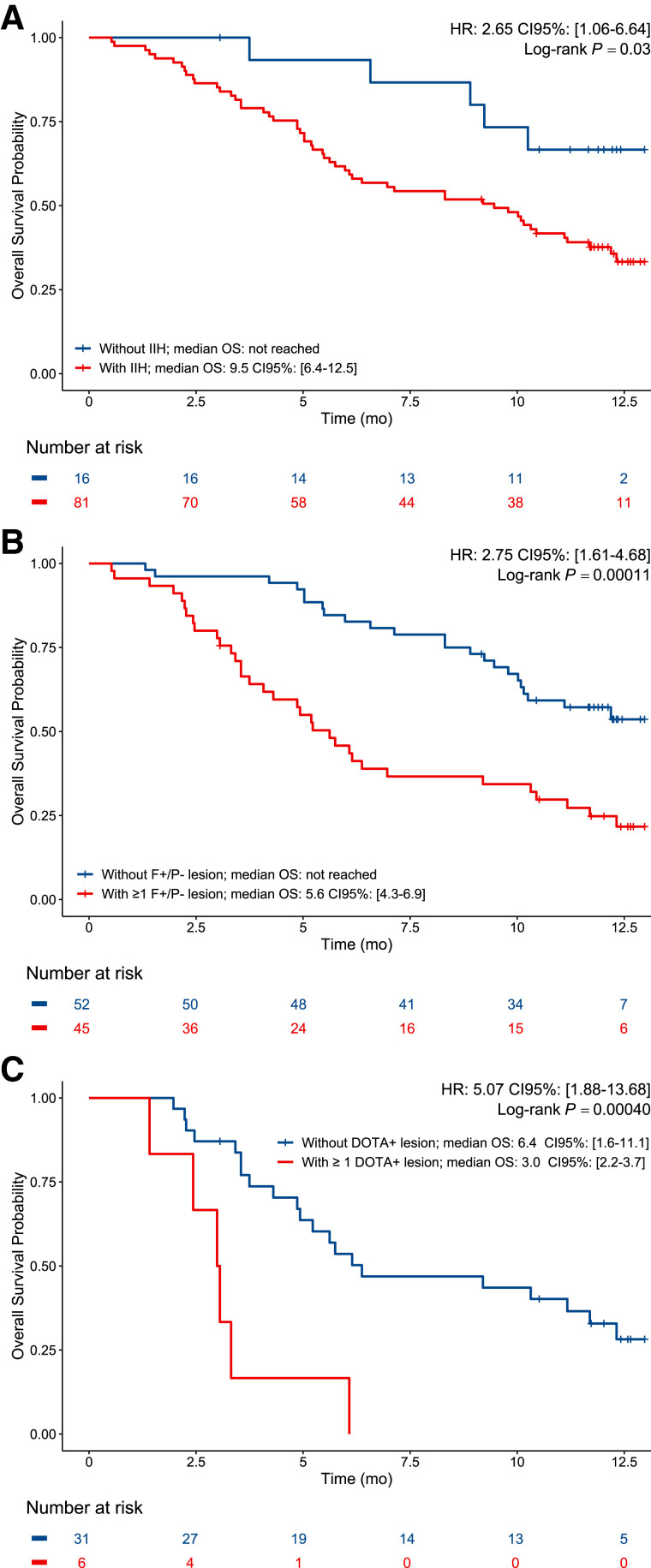
Association of OS with IIH and specific imaging phenotypes. (A) OS of patients with or without IIH. (B) OS of patients with or without ≥1 ^18^F-FDG+/^68^Ga-PSMA− lesion. (C) OS among patients who had ≥1 ^18^F-FDG+/^68^Ga-PSMA− lesion and underwent ^68^Ga-DOTATATE PET (*n* = 37) with or without ≥1 ^68^Ga-DOTATATE+ lesion.

The median OS of the 98 enrolled patients was 10.2 mo (95% CI, 8.5–11.8 mo). As shown in Figure 4A, patients with IIH had a statistically significant shorter median OS than patients without IIH: 9.5 mo (95% CI, 6.4–12.5 mo) versus not reached (log-rank *P* = 0.03; HR, 2.7; 95% CI 1.1–6.8). Moreover, patients with at least 1 ^18^F-FDG+/ ^68^Ga-PSMA− lesion had a shorter median OS than those without: 5.6 mo (95% CI, 4.3–6.9 mo) versus not reached (log-rank *P* = 0.0001; HR, 2.7; 95% CI, 1.6–4.7; Fig. 4B). Finally, in patients who underwent ^68^Ga-DOTATATE PET/CT (*n* = 37), the presence of at least 1 ^68^Ga-DOTATATE+ lesion was associated with a shorter median OS: 3.0 mo (95% CI, 2.2–3.7 mo) versus 6.4 mo (95% CI, 1.6–11.1 mo) (log-rank *P* = 0.0004; HR, 5.1; 95% CI, 1.9–13.7; Fig. 4C).

### Eligibility for PSMA or DOTATATE RPT

On the basis of the prespecified PET criteria, 52 of the 98 participants (53.1% [95% CI, 41.7%–62.2%]) would have been deemed eligible for PSMA RPT, and none (0%) for DOTATATE RPT. In other words, the standalone PSMA RPT would have allowed targeting all lesions of a significant volume (≥1 cm^3^ metabolic tumor volume with any PET tracer) and hypermetabolism (^18^F-FDG SUV_peak_ ratio ≥ 1.5) because the ^68^Ga-PSMA uptake of these lesions would have been significantly above that of the liver (SUV_peak_ ratio ≥ 1.5).

## DISCUSSION

Several studies have confirmed and molecularly characterized IIH in prostate cancer, but data on the prevalence of IIH considering the whole-body metastatic burden and especially at earlier stages of mCRPC are lacking (11,12,19,20). In opposition to tissue sampling, which assesses very few tumors (19), multitracer molecular imaging allows noninvasive whole-body tumor assessment and can identify different tracer uptake patterns among several metastases that may inform on their respective biology, guide biopsy, or select treatments such as RPT and intensification regimens. Pioneering imaging studies led by Hofman et al. on the prevalence of ^18^F-FDG+/^68^Ga-PSMA− lesions among patient candidates for PSMA RPT after chemotherapy paved the way for our study (10,21). Although they did not describe IIH specifically, they reported an 18% prevalence of patients harboring at least 1 ^18^F-FDG+/^68^Ga-PSMA− lesion in CRPC patients after androgen receptor-pathway inhibitors and docetaxel, using a different PSMA positivity threshold from the one we used (SUV_max_ of 10) (10). Others have reported an IIH prevalence of approximately 25% based on the presence of at least 1 ^18^F-FDG+/^68^Ga-PSMA− lesion in CRPC patients, but with few or no lines of systemic therapy (22,23). Our study is distinct because we thoroughly characterized IIH using prespecified quantitative PET criteria that enabled us to distinguish ^68^Ga-PSMA+-only, ^18^F-FDG+-only, and ^18^F-FDG+/^68^Ga-PSMA+ lesions. One key finding is that 6% of our participants had exclusively ^18^F-FDG+/^68^Ga-PSMA− disease, that is, no ^68^Ga-PSMA+ disease, a prevalence similar to the 10% rate found in the TheraP study (10,24). This subgroup is of the utmost importance because these patients will probably have a distinct tumor biology and prognosis and are not candidates for PSMA RPT (14,25,26). Interestingly, we found that clinical factors associated with patients harboring at least 1 ^18^F-FDG+/^68^Ga-PSMA− lesion were an increasing number of lines of systemic therapy received and the presence of visceral metastasis on conventional imaging (Supplemental Table 4). These factors can be added to those reported previously in patients with fewer lines of therapy against CRPC such as International Society of Urologic Pathologists grade, prostate-specific antigen value, or prostate-specific antigen doubling time (10,22–24). The combination of these factors may help clinicians to decide whether a patient should undergo ^18^F-FDG PET/CT during PSMA RPT eligibility evaluation.

Another key finding is that 82.7% of patients had more than one imaging phenotype based on double-tracer imaging, defining an unexpected high level of IIH in mCRPC. This high prevalence of IIH correlates with a recently published paper that reported an IIH prevalence of 32% and 36% based on PSMA- and STEAP1-positive or -negative immunostainings, respectively, after analysis on an average of only 3 samples per patient (27). Because IIH unveils several biologies in the same individual, patients with IIH might benefit most from treatment intensification combinations compared with single-agent regimes, a strategy shown to prolong time to progression in both hormone-sensitive prostate cancer and CRPC patients (28–32). In our study, OS was different depending on the presence of a specific imaging phenotype (Fig. 4). Patients harboring at least 1 ^18^F-FDG+/^68^Ga-PSMA− or at least 1 ^68^Ga-DOTATATE+ lesion had decreased OS. In a smaller cohort of patients (*n* = 17) after 1 line of treatment, Bilen et al. also found that ^68^Ga-DOTATATE uptake was associated with shorter OS, whereas Iravani et al. reported in 5 patients with CRPC NED that none were candidates for DOTATATE RPT (33,34). ^68^Ga-DOTATATE PET imaging could therefore help clinicians in deciding whether to use another line of therapy versus palliative care because of the very poor outcomes associated with ^68^Ga-DOTATATE positivity (median, 3.0 mo). However, candidacy for DOTATATE RPT seems to be rare in CRPC, even in patients with CRPC NED.

Our study had limitations. First, the dichotomization of uptake as positive or negative does not fully exploit the continuous nature of PET metrics and may have contributed to increasing the apparent prevalence of IIH. However, we observed opposed phenotypes in 21% of participants (e.g., both ^18^F-FDG+/^68^Ga-PSMA− and ^18^F-FDG−/^68^Ga-PSMA+), which we interpreted as evidence of marked IIH in a substantial subset of our population. On the other hand, the choices of positivity threshold, minimum tumor volume, and maximum number of lesions analyzed were guided by both clinical and practical considerations and were intentionally biased toward specificity by excluding the smallest lesions and those with mild uptake. Although one could argue that we did not characterize the total number of potential lesions for many participants, doing so would have actually increased the apparent prevalence of IIH by introducing apparent lesions for which the clinical relevance or specificity could be questioned. We elected to mandate ^68^Ga-DOTATATE PET/CT in only a subset of our cohort for financial and logistic reasons, as well as to avoid multiple scans with an uncertain direct benefit to individual patients. The counterpart is that we could not compare triple-tracer imaging in all of our patients, which probably would have highlighted other combinations of phenotypes.

## CONCLUSION

We found that IIH is highly prevalent in mCRPC patients. The high prevalence of IIH, the plurality of the phenotypic combinations, and the exploratory OS data highlight the importance of further characterizing the molecular and prognostic significance of each imaging phenotype to better select patients for intensified management and guide biopsy sites for precision medicine.

## DISCLOSURE

The study was supported by ONCOPOLE EMC2, a peer-reviewed funding grant that matched funds from the Fonds de Recherche du Québec–Santé public agency, the Cancer Research Society, and Merck Canada Inc. No representatives from these organizations were involved in the designer data analysis of this study. The principal investigators and their collaborators are solely responsible for the data, conduct of the study, and publication of results. An in-kind contribution was received from Hermes Medical Solutions Canada for free access to its imaging transfer platform. Frédéric Pouliot, Etienne Rousseau, Patrick Richard, and Jean-Mathieu Beauregard are supported by Fonds de recherche du Québec–Santé as clinical research scholars. No other potential conflict of interest relevant to this article was reported.

## Notes

KEY POINTS **QUESTION:** How prevalent is IIH in mCRPC patients? **PERTINENT FINDINGS:** This study on 98 mCRPC patients showed that IIH has a prevalence of 82.7% and is associated with a shorter median OS. Twelve different combinations of lesion imaging phenotypes based on triple ^18^F-FDG, ^68^Ga-PSMA, and ^68^Ga-DOTATATE PET/CT were found among patients. **IMPLICATIONS FOR PATIENT CARE:** A multitracer imaging strategy can be exploited to guide biopsy and personalize management of advanced prostate cancer.
